# Exploring the Antiviral Potential of *Artemisia annua* Through JAK-STAT Pathway Targeting: A Network Pharmacology Approach

**DOI:** 10.3390/ph17111539

**Published:** 2024-11-16

**Authors:** Mebarka Ouassaf, Lotfi Bourougaa, Farial Bahaz, Bader Y. Alhatlani

**Affiliations:** 1 Group of Computational and Medicinal Chemistry, LMCE Laboratory, University of Biskra, BP 145, Biskra 07000, Algeria; lotfi.bourougaa@univ-biskra.dz; 2 Laboratory of Organic Materials and Heterochemistry, Echahid Cheikh Larbi Tebessi University, Tebessa 12000, Algeria; farial.bahaz@univ-tebessa.dz; 3 Unit of Scientific Research, Applied College, Qassim University, Buraydah 52571, Saudi Arabia

**Keywords:** antiviral, *A. annua*, network pharmacology, JAK-STAT, molecular dynamics simulations, FEL

## Abstract

Background: *Artemisia annua*, a plant with antiviral potential, has shown promise against various viral infections, yet its mechanisms of action are not fully understood. This study explores *A. annua*’s antiviral effects using network pharmacology and molecular docking, focusing on key active compounds and their interactions with viral protein targets, particularly within the JAK-STAT signaling pathway—a critical mediator of immune responses to viral infections. Methods: From the TCMSP database, we identified eight active compounds and 335 drug targets for *A. annua*, with 19 intersecting targets between *A. annua* compounds and viral proteins. A protein–protein interaction (PPI) network highlighted 10 key hub genes, analyzed further through Gene Ontology (GO) and KEGG pathways to understand their immune and antiviral roles. ADMET properties of the active compound Patuletin (MOL004112) were assessed, followed by 200 ns molecular dynamics simulations to examine its stability in complex with JAK2. Results: PPI analysis identified JAK2, MAPK3, MAPK1, JAK1, PTPN1, HSPA8, TYK2, RAF1, MAPT, and HMOX1 as key hub genes, with JAK2 emerging as a critical regulator of immune and antiviral pathways. ADMET analysis confirmed Patuletin’s favorable pharmacokinetic properties, and molecular dynamics simulations showed a stable Patuletin-JAK2 complex, with FEL analysis indicating minimal disruption to JAK2’s intrinsic flexibility. Conclusions: These findings highlight JAK2 as a promising target in the antiviral activity of *A. annua* compounds, particularly Patuletin, supporting its potential as an antiviral agent and providing a foundation for further research on *A. annua*’s therapeutic applications.

## 1. Introduction

Viral infections continue to pose a major global public health threat, exacerbated by factors such as globalization, climate change, and socioeconomic disparities. In 2021 alone, poverty-related infectious diseases (vIDPs) were responsible for an estimated 8.7 million deaths and 259.2 million disability-adjusted life years (DALYs) worldwide [1]. The search for new antiviral therapies is more urgent than ever, as existing treatments struggle to keep up with evolving viral pathogens. The emergence of viral strains resistant to current antivirals complicates the management of pandemics, as observed with HIV, the hepatitis C virus, and more recently, SARS-CoV-2. This resistance highlights the importance of finding new molecules capable of targeting different mechanisms in the viral cycle [2]. One promising area of exploration is the antiviral properties of medicinal plants, which have been traditionally used across cultures for centuries. Medicinal plants represent a vast and still largely untapped reservoir of bioactive compounds. Thanks to their ability to synthesize a wide variety of biochemical molecules, they have enabled the discovery of essential drugs such as quinine and morphine. The continued exploration of plant biodiversity is therefore crucial for identifying new antiviral treatments [3]. These plants are often rich in bioactive compounds capable of inhibiting viral replication and modulating the immune response, offering a valuable alternative for developing novel antiviral treatments.

Among these, *Artemisia annua* has gained significant attention due to its bioactive compounds, particularly artemisinin, which has demonstrated broad-spectrum antiviral properties [4,5,6,7]. *A. annua* has been shown to interfere directly with viral replication and modulate host immune responses, making it effective against viruses such as influenza, HIV, hepatitis B, and SARS-CoV-2 [8,9,10,11]. This highlights the importance of identifying active compounds and pathways through which *A. annua* exerts these antiviral effects.

In silico approaches, such as molecular docking and molecular dynamics simulations, can reduce the time and cost of drug discovery. They provide rapid and accurate predictions of ligand–receptor interactions, facilitating the screening of the most promising compounds before moving to experimental testing [12].

Given the complexity of plant-based compounds and their interactions with biological systems, network pharmacology offers a valuable approach to understanding these interactions. This method explores how bioactive compounds affect multiple biological targets and pathways simultaneously, providing a comprehensive view of their therapeutic potential [13,14,15,16]. By applying network pharmacology to *A. annua*, we can map the interactions between its bioactive compounds and relevant biological targets involved in viral infections. This enables us to identify key protein targets and understand the mechanisms by which *A. annua* compounds exert their antiviral effects.

In this study, we employ network pharmacology and molecular docking to explore the antiviral potential of *A. annua*. After identifying key protein targets, we assess their binding interactions with *A. annua* compounds through docking simulations. Furthermore, we integrate ADMET (Absorption, Distribution, Metabolism, Excretion, and Toxicity) and molecular dynamics analyses to gain deeper insights into the pharmacokinetics and stability of these interactions. This multifaceted approach aims to uncover the therapeutic potential of *A. annua* against viral infections and lay the groundwork for future antiviral drug development.

## 2. Results

### 2.1. Common Targets of A. annua and Antiviral Agents

**Figure 1:**
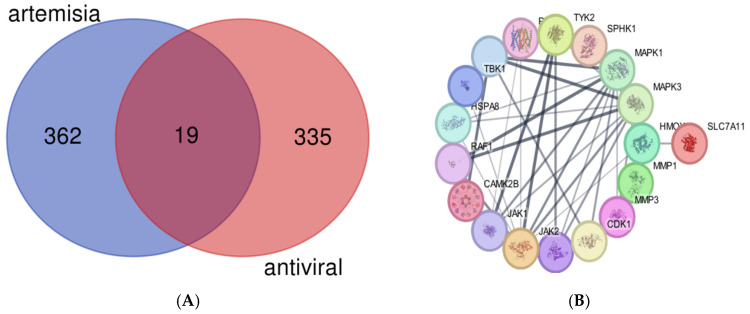
(**A**) Venn diagram showing the overlap between the *A. annua* target genes and antiviral-related target genes, identifying the 19 common targets. (**B**) Protein–protein interaction (PPI) network of target genes associated with *A. annua* compounds, visualized using data from the STRING database.

To identify the active compounds derived from *A. annua*, we selected compounds from TCMSP and applied specific criteria to determine their suitability for drug development, as outlined in Section 4.1. According to our selection approach, compounds with an oral bioavailability (OB) greater than 30% and a drug-likeness (DL) greater than 0.18 are considered appropriate candidates. Using these criteria, along with a molecular mass range of 250 to 500 Da, we filtered the compounds to ensure they met these standards. The selected compounds, meeting OB ≥ 30% and DL ≥ 0.18, are presented in Table 1 (see also Figure S1). After identifying the target genes for each of the eight active compounds derived from *A. annua* and removing duplicates and non-protein-coding genes, we identified 366 potential targets for *A. annua* and 335 potential antiviral targets (Supplementary Table S1). A Venn diagram, generated using the online tool available at https://bioinformatics.psb.ugent.be/webtools/Venn/ (accessed on 20 September 2024), was employed to visualize the intersection of these target sets. This analysis revealed 19 common targets between *A. annua* and antiviral-related compounds, as depicted in Figure 1A.

The Protein–Protein Interaction (PPI) data was retrieved from the STRING database (https://string-db.org/, accessed on 15 July 2024), which assigns confidence scores to interactions categorized as high (>0.7), medium (>0.4), and low (>0.15). For this study, we constructed our PPI network using a confidence threshold of >0.4 and restricted it to the species *Homo sapiens.* The initial PPI network from STRING is illustrated in Figure 1B. Subsequently, the PPI data obtained from STRING was imported into Cytoscape (https://apps.cytoscape.org/apps/cytohubba, accessed on 3 October 2024) for further visualization and analysis of the network interactions.

### 2.2. Gene Ontology and KEEG Pathway Analysis

An enrichment analysis was conducted to identify the relevant domains, pathways, and gene ontologies (GO) associated with the 19 common genes. Using the DAVID database, we categorized these genes into three GO functional categories: Biological Processes (BP), Cellular Components (CC), and Molecular Functions (MF). To further visualize and interpret the data, bar charts were generated using the bioinformatics platform https://www.bioinformatics.com.cn/en, accessed on 23 October 2024).

**Figure 2:**
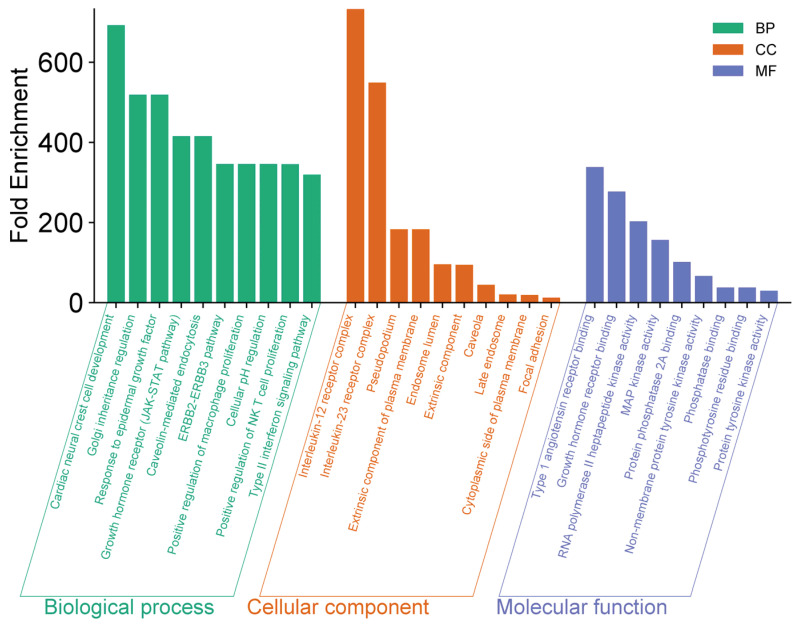
GO enrichment analysis of 19 target genes associated with *A. annua* antiviral activity.

The results of the GO enrichment analysis are shown in Figure 2, highlighting the involvement of these genes in key antiviral biological processes. The enriched biological processes include cardiac neural crest cell development, macrophage proliferation, and NK cell proliferation, all of which are crucial for modulating the antiviral immune response.

In terms of Cellular Components (CC), the IL-12/23 receptor complex and plasma membranes were notably enriched, suggesting their roles in mediating immune cell signaling and viral recognition.

For Molecular Functions (MF), the enrichment analysis indicated a significant presence of kinase activity, particularly tyrosine kinase activity, which may play a pivotal role in the regulation of antiviral signaling pathways. This further suggests that the interaction of *A. annua* compounds with these molecular functions could enhance antiviral defense mechanisms.

### 2.3. Construction of a PPI Network and Hub Gene Selection

**Figure 3:**
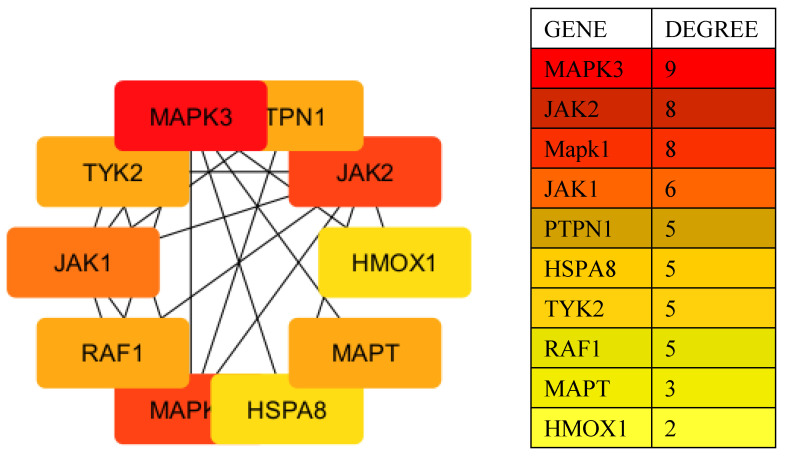
Top 10 genes ranked by their degrees of interaction in the protein–protein interaction (PPI) network. The hub genes identified include MAPK3, JAK2, MAPK1, JAK1, PTPN1, HSPA8, TYK2, RAF1, MAPT, and HMOX1. The size and color of the nodes reflect the degree of interactions, with MAPK3 highlighted in red as the most highly connected gene, indicating its central role in the network.

Using Cytoscape software 3.10.23.10.2 [12], a protein–protein interaction (PPI) network was constructed based on data from STRING 12.0. The CytoHubba plug-in (https://apps.cytoscape.org/apps/cytohubba, accessed on 3 October 2024) was utilized to identify the top hub genes with the highest degree of interactions. As shown in Figure 3, these hub genes were MAPK3, JAK2, MAPK1, JAK1, PTPN1, HSPA8, TYK2, RAF1, MAPT, and HMOX1.

The PPI network highlights the core proteins and their interactions, emphasizing the central role of MAPK3/ERK, which is marked in red to signify its high degree of interactions. MAPK3 serves as a crucial protein within this network, directly interacting with other key proteins, such as JAK2, RAF1, and TP53, which are integral to important signaling pathways, including the MAPK/ERK and JAK-STAT pathways.

Among the other hub genes, JAK2 also plays a prominent role, with numerous interactions reflecting its involvement in the JAK-STAT signaling pathway, which is essential for immune response and cell proliferation. Additionally, proteins such as PTPN1 and HMOX1 show moderate to lower degrees of interaction (colored orange to yellow) but still hold significant biological importance. PTPN1 is involved in signaling pathways by interacting with MAPK3 and other proteins, while HMOX1 is a key player in heme degradation and cytoprotection.

HSPA8, marked in yellow, is a chaperone protein crucial for protein folding and cellular stress response, indicating the importance of these processes in maintaining cellular homeostasis. This overall network of interactions underscores the biological significance of pathways like MAPK/ERK and JAK-STAT, with proteins such as MAPK3, RAF1, and JAK2 serving as potential therapeutic targets for diseases in which these signaling pathways are dysregulated, such as cancer and inflammatory diseases.

### 2.4. GO Enrichment Analysis

**Figure 4:**
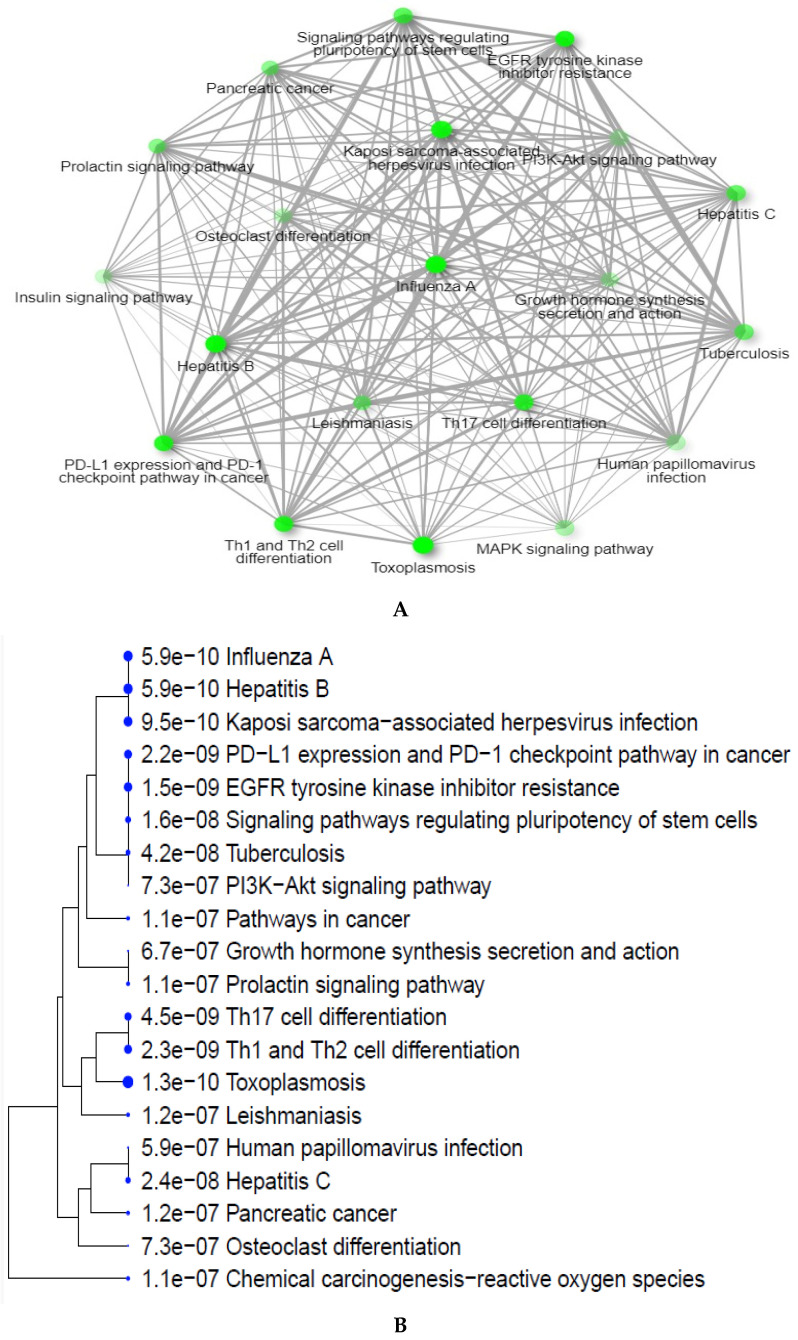

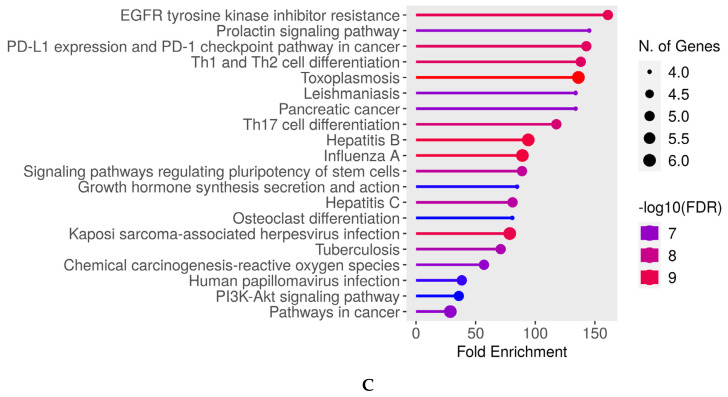
Enrichment analyses of targeted genes: (**A**) KEGG pathway analysis showing the involvement of the top 10 hub genes in critical biological pathways related to viral infections, immune response, and cell signaling. (**B**) Clustering analysis illustrating the relationships and groupings between enriched pathways, highlighting connections between immune system regulation, oncogenic processes, and inflammation. (**C**) Pathways interaction network, visualizing the interplay between multiple signaling pathways, such as the MAPK/ERK and JAK-STAT pathways, showcasing how these pathways interact with each other and contribute to disease progression and immune response regulation.

GO enrichment analysis was performed using the Shiny GO tool 0.80 to assess the biological pathways and molecular functions related to the 10 hub genes. The results, shown in Figure 4A–C and Table 2, provide valuable insights into the antiviral mechanisms of *A. annua*. In Figure 4A, the network of interactions highlights the connection between viral infections and key cellular signaling pathways, such as PI3K-Akt and PD-L1/PD-1. These pathways are essential for modulating immune responses and inflammation, which also play a role in cancer progression, cell differentiation, and immune regulation, demonstrating the broad influence of these pathways on viral and immune-related processes.

Figure 4B presents a gene enrichment analysis showing the involvement of hub genes in pathways related to major viral infections, such as hepatitis B, hepatitis C, influenza A, and Kaposi’s sarcoma herpesvirus. These pathways are closely associated with both antiviral responses and oncogenic processes, emphasizing the dual role of viral infections in cancer development and immune response regulation. For example, human papillomavirus (HPV) is strongly linked to cervical cancer, illustrating how viral infections can contribute to immune system challenges and cancer progression. The figure includes adjusted *p*-values (Shiny GO FDR) for each pathway, reflecting the likelihood that these associations are not due to random chance. The exponential notation (e.g., 1.1 × 10^−7^) highlights pathways strongly enriched for hub genes, with lower values close to zero signifying high statistical significance. This underscores the relevance of these pathways in viral infections and their critical roles in both immune response and oncogenesis. Similarly, Figure 4C offers a bar graph illustrating the enrichment of the signaling pathways involved in viral infections, particularly those related to immune system regulation, proliferation, and differentiation. These pathways underscore the broader implications of viral infections in promoting inflammatory and oncogenic diseases. In Table 2, key genes such as JAK1, JAK2, MAPK1, and RAF1 are identified as being enriched in these viral infection pathways. The recurrent presence of JAK2, especially in the hepatitis B and influenza A pathways, highlights its pivotal role and positions it as a key target for future molecular docking studies aimed at exploring the molecular interactions between *A. annua* compounds and this protein. This analysis reinforces the therapeutic potential of *A. annua* in treating viral infections by modulating key signaling pathways involved in immune response and viral pathogenesis.

### 2.5. Docking Analysis of A. annua-Derived Compounds as JAK2 Inhibitors

In this study, we selected the hub gene JAK2 from the 10 identified genes for docking analysis with eight active compounds derived from *A. annua*. JAK2 is a member of the Janus kinase (JAK) family, which is crucial for cytokine signaling and the regulation of various cellular responses, including hematopoiesis and immune functions. Specifically, JAK2 is involved in cytokine receptor signal transduction and is associated with several pathologies, such as myeloproliferative disorders [17,18]. Our goal is to explore the interactions between JAK2 and the active compounds of *A. annua*, focusing on their potential inhibitory effects, to enhance our understanding of the mechanisms underlying the plant’s therapeutic utility.

The 3D structure of JAK2 (PDB ID: 7F7W) was obtained from the Protein Data Bank (PDB) (https://www.rcsb.org/structure/7F7W, accessed on 10 October 2024). This protein structure was selected due to its high resolution (1.83 Å) and previous studies conducted on it [19,20], which reinforce the reliability of our analysis. The docking procedures, detailed in Section 4.5, emphasized the importance of ensuring that the compounds interact correctly with the target protein’s active site. This active site was identified based on the position of the reference compound 36H, which was already docked in the crystal structure. The key amino acids constituting the active site include Val629, Lys581, Leu551, Gln553, Ser755, Gly554, Thr555, Phe556, Thr557, Lys558, Ile559, Leu579, Asp584, His587, Phe594, Gln626, Glu627, Phe628, Leu549 Val629 Leu680 Leu551, Lys630, Phe631, Gly632, Ser633, Asp635, Thr636, Asn673, Val674, Cys675, Ala676, Lys677, Ile559. and Asn678.

**Figure 5:**
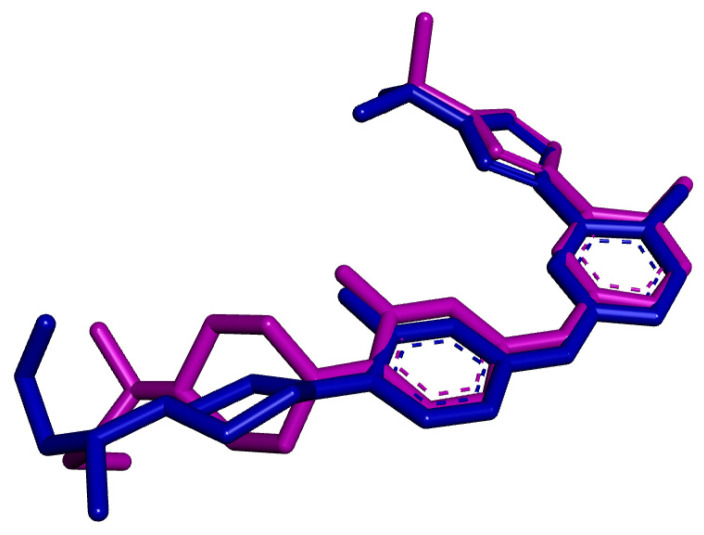
Superposition of the reference compound (pink) after redocking with the original crystallized compound (blue) in the protein complex.

To ensure the reliability of our docking methodology, we first sought to validate its accuracy by comparing the predicted binding affinities of known JAK2 inhibitors (Table S2 depicts their experimentally determined Ki values). The correlation plot (Figure S2) illustrates the relationship between the calculated docking scores and the experimental Ki values for a series of known JAK2 inhibitors. We generated this plot using compounds obtained from BindingDB (https://www.bindingdb.org/rwd/bind/AdvancedSearch.jsp, accessed on 6 November 2024). The observed trend generally showed an inverse correlation, where lower docking scores (indicating stronger predicted binding affinities) corresponded to lower K_i values, which aligns with higher binding affinities experimentally. This consistency suggests that the docking protocol is effective in predicting the binding strengths for JAK2 inhibitors. However, some deviations from the expected trend were observed for specific compounds, which may be due to structural variations or limitations in the scoring function. Overall, this analysis provides further support for the validity of our computational approach. As a next step in the validation process, we performed a second validation method, which involved redocking the reference compound and calculating the root mean square deviation (RMSD) to assess the reliability and accuracy of our predictions. We performed docking with the reference compound and compared the oot ean quare eviation (RMSD) to our results and the original compound’s position in the crystal structure (Figure 5 and Figure S3). An RMSD value of less than 3 Å is indicative of reliable results [21]. In our study, the calculated RMSD was 2.073 Å, which is well below the threshold, indicating that our predicted position closely matches that of the reference compound in the active site. This reinforces the reliability of our docking results, which are essential for assessing the efficacy of the compounds under investigation.

Here, we compared the docking scores of the compounds not only with H36, the ligand that is co-crystallized with the JAK2 protein and is used as a reference to understand the binding properties of compounds that interact with JAK2 in a similar manner (Figure S4), but also with the approved JAK2 inhibitor, Ruxolitinib (Figure S5). Ruxolitinib, a clinically approved JAK2 inhibitor, was used as a positive control to assess the efficacy of the new compounds against the established drug.

**Figure 6:**
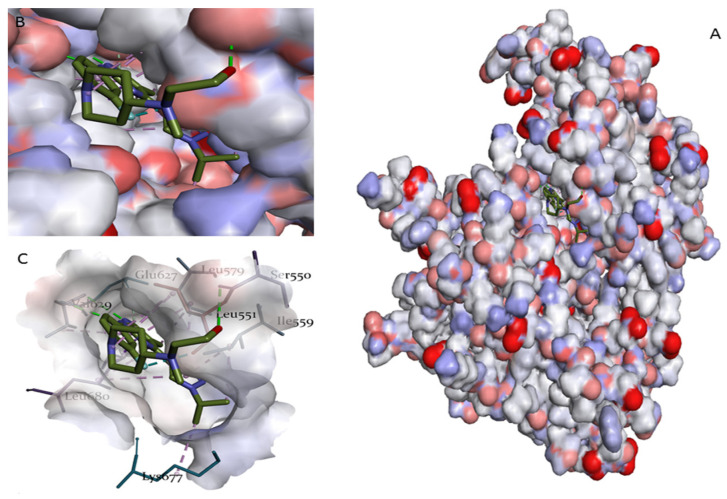
Surface representation of JAK2 bound to the reference compound H36: (**A**) Overall surface view of JAK2 with bound H36. (**B**) Close-up of active site interactions between JAK2 and H36. (**C**) distribution of surface forces and hydrogen bond interactions stabilizing H36 in JAK2.

A key observation from the interaction analysis of the complexes is that the amino acids interacting with our compounds, the reference compound, and Ruxolitinib were quite similar, lending support to the validity of our study. Further analysis of the 2D and 3D interaction forms (Figure 6) revealed two main types of interactions between the *A. annua* compounds and the JAK2 protein in its active site: hydrogen bonds and hydrophobic interactions, which were also present in the interactions with compound H36 and Ruxolitinib. Hydrophobic interactions, where non-polar regions of molecules aggregate to avoid water, play a critical role in stabilizing the protein–ligand binding [22].

Figure 6 illustrates the interaction between the JAK2 enzyme and the reference compound H36 through a three-dimensional surface representation. Panel A displays the overall surface of JAK2 bound to H36, providing an overview of the binding site and the distribution of amino acids on the surface. Panel B reveals details of the active site interactions, where H36 engages with critical amino acid residues in JAK2, enhancing molecular stability through hydrogen bonds and hydrophobic interactions. Finally, Panel C highlights the stabilizing surface forces, including hydrogen bonds and hydrophobic interactions, which support the positioning of H36 within the active site.

The results indicate that MOL002235 and MOL004112 achieved higher binding energy scores of −10.506 kcal/mol and −9.570 kcal/mol, respectively, as compared to H36 (−9.432 kcal/mol) and Ruxolitinib (−6.829 kcal/mol) (Table 3).

**Figure 7:**
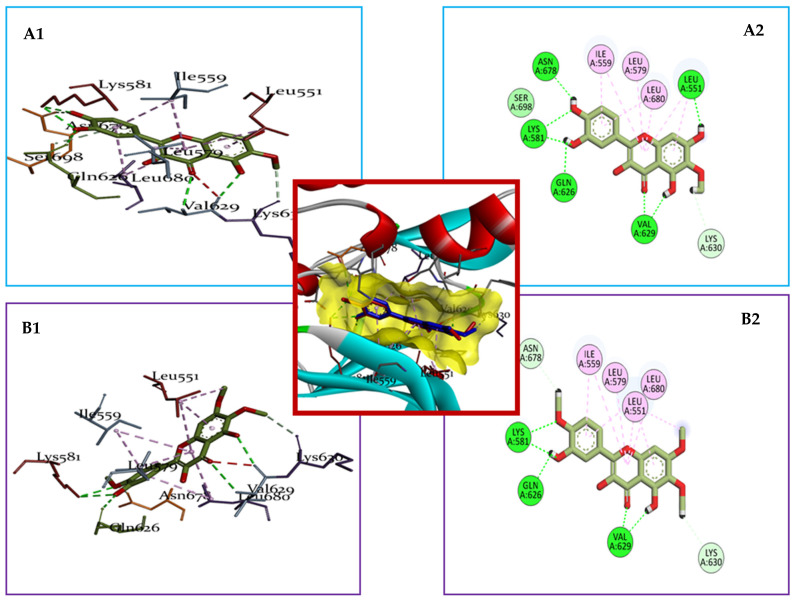
3D and 2D visualization of the binding interactions of compounds (**A1**,**A2**) (MOL004112) and (**B1**,**B2**) (MOL002235) with the JAK2 protein (PDB: 7F7W). The figure illustrates the specific interactions between the compounds and key residues within the active site of JAK2, highlighting both the hydrogen bonds and hydrophobic interactions.

The superior performance of MOL002235 and MOL004112 can be attributed to the diversity and abundance of their hydrophobic interactions, as well as a higher number of hydrogen bonds with key residues of JAK2 (Figure 7). Specifically, these compounds interact hydrophobically with residues such as Ile559, Leu572, Leu551, and Leu680, while forming a significant number of stable hydrogen bonds (approximately 2.7 Å) with residues like Lys581, Gln626, Val629, Lys630, and Asn678 (Table 4). In contrast, Ruxolitinib and H36 exhibit fewer hydrogen interactions that are limited to residues Thr636, Gln626, and Glu627. The increased number of hydrogen bonds in MOL002235 and MOL004112 reinforces their stabilities, which likely accounts for their superior performances as potential JAK2 inhibitors. Hydrogen bonds play a crucial role in the stability and specificity of ligand–protein interactions. These bonds, although weaker than covalent bonds, contribute significantly to the proper alignment and binding of the ligand to the protein’s active site, enhancing the stability of the complex. A higher number of hydrogen bonds often results in a stronger and more stable binding, which is critical for the effectiveness of inhibitors like MOL002235 and MOL004112.

### 2.6. ADMET Profile of A. annua Compounds

The ADMET analysis of the *A. annua* compounds reveals several noteworthy properties that support their potential as JAK2 inhibitors for viral treatment. The results presented in Table 5, Table 6 and Table 7 indicate that both MOL004112 and MOL002235 exhibit favorable characteristics regarding pharmacokinetics and toxicity, albeit with some notable differences as compared to the reference drug Ruxolitinib.

Table 5 highlights the molecular properties of the compounds, showing that MOL002235 has good solubility and low lipophilicity (LogP = 1.777), suggesting excellent absorption and satisfactory oral bioavailability—key attributes for an antiviral drug targeting JAK2. In contrast, MOL004112 displays slightly higher polarity (TPSA = 140.59), which may affect its ability to traverse cell membranes. This increased polarity could enhance its interaction with enzymatic targets, particularly JAK2, in hydrophilic environments, thereby potentially improving its therapeutic efficacy.

Table 6 highlights the drug-likeness of the compounds, revealing that MOL002235, with a Quality by Design (QED) score of 0.65, is more favorable for drug development. Importantly, none of the compounds trigger PAINS alerts, which is crucial for avoiding interference in biological assays. Both compounds also comply with Lipinski’s rule, suggesting their good absorption potential following oral administration.

The data presented in Table 7 and Table S4 indicate that both MOL004112 (Patuletin) and MOL002235 exhibit favorable intestinal permeability, which is crucial for achieving satisfactory bioavailability after oral administration. In addition, these compounds show no immediate risks of toxicity, including hepatic, neurotoxic, or mutagenic effects, reinforcing their potential as safe JAK2 inhibitors for viral infection treatment.

In terms of volume of distribution (Vd), both compounds display relatively low values, suggesting limited tissue distribution. This characteristic may be advantageous for drugs targeting specific enzymes like JAK2, as a lower Vd helps to concentrate the drug’s effects on the target site and may also reduce the risk of systemic toxicity.

The ADMET analysis highlights both the promising and challenging aspects of these compounds. For instance, Patuletin demonstrates excellent intestinal absorption (HIA), suggesting strong bioavailability when taken orally. However, its high plasma-protein binding (PPB) value of 97.7% indicates that a significant portion of the compound could be bound to plasma proteins, potentially limiting the amount of active, free compound in circulation. By comparison, Ruxolitinib—a clinically approved JAK inhibitor—has a PPB of 56%, allowing a larger proportion of free compound in the bloodstream, which may contribute to its therapeutic effectiveness. This comparison implies that, despite Patuletin’s elevated PPB, it could still have therapeutic potential, especially if dosing strategies or formulations are optimized to enhance its bioavailability.

Regarding metabolism (Table S3), MOL002235 inhibits several cytochrome P450 enzymes, including CYP1A2 and CYP2C19. This inhibition raises concerns about potential drug–drug interactions, an important consideration during the development of this compound. It is essential to evaluate these interactions to ensure the safety and efficacy of the treatment.

Table S4 on toxicity reveals that all the compounds studied, including MOL004112, MOL002235, and the reference drug Ruxolitinib, exhibit favorable toxicity profiles. Specifically, MOL004112 and MOL002235 do not show any hepatic, neurotoxic, immunotoxic, mutagenic, or cytotoxic effects, and they are unable to cross the blood–brain barrier (BBB), which is advantageous for developing JAK2 inhibitors for viral infection treatment.

In contrast, Ruxolitinib presents some risk of neurotoxicity and crosses the BBB, potentially limiting its use in certain clinical contexts. These findings underscore the interest in further developing the compounds derived from *A. annua* due to their superior toxicity profiles.

MOL004112 was selected for further study (Table 8) because of its specific properties, which suggest a more favorable pharmacokinetic and pharmacodynamic profile. This compound may offer improved interaction with JAK2 and a reduced risk of inhibiting cytochrome P450 enzymes, thereby limiting the chances of drug–drug interactions. Additionally, MOL004112 may have shown better stability or efficacy in preliminary in silico studies, justifying its selection as a lead candidate for future antiviral research targeting JAK2.

### 2.7. Molecular Dynamic Simulation (MDS)

**Figure 8:**
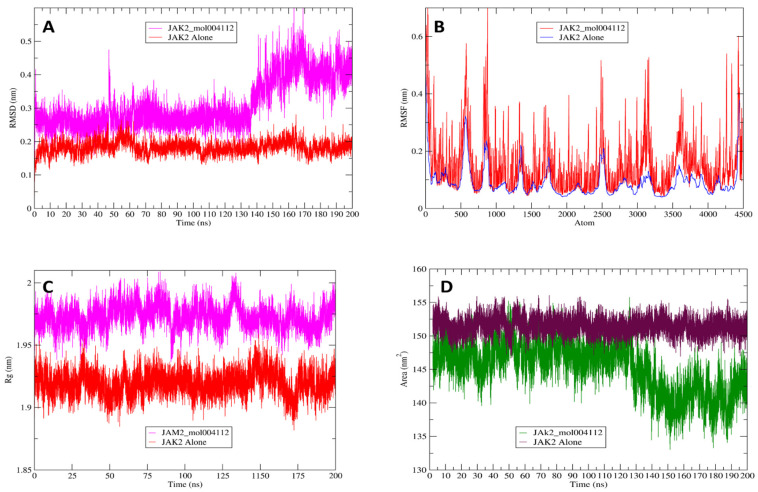
Biomolecular structure evaluation: (**A**) RMSD (root mean square deviation) values comparing the stability of the JAK2-MOL004112 complex and JAK2 alone throughout the molecular dynamics simulation. (**B**) RMSF (root mean square fluctuation) analysis depicting the flexibility of individual amino acid residues in the JAK2-MOL004112 complex as compared to JAK2 alone. (**C**) Radius of gyration (Rg) illustrating the compactness of the JAK2-MOL004112 complex relative to JAK2 alone. (**D**) Solvent Accessible Surface Area (SASA) highlighting the exposure of the hydrophilic and hydrophobic residues in the JAK2-MOL004112 complex as compared to JAK2 alone.

The optimal docked conformation of MOL004112 with the JAK2 enzyme was further evaluated through molecular dynamics (MD) simulations to assess its thermodynamic properties and stability. For comparison, the JAK2 protein was independently simulated under identical conditions. The root mean square deviation (RMSD) was used to measure the structural stability of the JAK2-ligand complex, with RMSD values lower than 2 Å indicating stability [29]. The RMSD of the JAK2 backbone was used to quantify the conformational stability of the protein–ligand complex throughout the simulation, as shown in Figure 8A. This metric reflects the degree of structural deviation over time, with lower RMSD values indicating that the complex maintains structural integrity and remains closer to its initial conformation, signifying higher stability. Conversely, higher RMSD values suggest greater conformational fluctuations, implying less stability and more significant deviations from the starting structure.

The average RMSD value for the JAK2-MOL004112 complex was measured at 0.300 nm, while the JAK2 protein alone exhibited an average RMSD value of 0.199 nm (Table 9). These results indicate that MOL004112 forms a stable complex with the JAK2 receptor, as the moderate increase in RMSD compared to the protein alone suggests that the ligand induces only minor conformational changes, thereby maintaining overall complex stability.

The RMSF (root mean square fluctuation) values for each amino acid residue in the protein backbone are depicted in Figure 8B. In this graph, the peaks correspond to the fluctuation of each residue throughout the simulation’s duration. Higher RMSF values indicate greater flexibility of the respective amino acid residues, suggesting regions of the protein that exhibit increased motion. In contrast, lower RMSF values reflect reduced flexibility, implying that those residues are more rigid and contribute to enhanced system stability. The average RMSF value for JAK2 backbone residues was 0.101 nm during the 200 ns simulation, while the average RMSF value for the JAK2-MOL004112 complex was 0.150 nm. These results suggest that the binding of MOL004112 slightly increases the flexibility of certain regions within the JAK2 backbone. However, the overall increase in flexibility remains moderate, indicating that the complex retains a stable conformation despite the interaction with the ligand.

The radius of gyration (Rg) serves as an indicator of the overall compactness and structural distribution of a molecule. The average Rg value for the JAK2-MOL004112 complex was 1.972 nm, as compared to 1.919 nm for JAK2 alone (Figure 8C). This minimal increase in Rg upon ligand binding indicates that the ligand does not induce significant conformational changes or cause the protein to unfold. Instead, the complex maintains a structurally compact and stable form, further suggesting that the ligand interaction does not destabilize or dramatically alter the overall architecture of the JAK2 receptor. The near similarity in Rg values implies that the JAK2 protein remains structurally consistent even in the presence of the compound MOL004112. These findings align with previous results from the RMSD and RMSF analyses, as the moderate changes in these values, coupled with the minimal increase in the radius of gyration, collectively indicate that the binding of MOL004112 to JAK2 does not significantly disrupt the structural stability or flexibility of the protein. This confirms that the protein–ligand complex remains stable throughout the simulation, maintaining a compact and well-structured conformation.

The solvent-accessible surface area (SASA) of both JAK2 alone and the JAK2-MOL004112 complex was evaluated to assess changes in the exposure of hydrophilic and hydrophobic residues during the molecular dynamics simulation. The findings, illustrated in Figure 8D, show that the average SASA values for the JAK2-MOL004112 complex (148.313 nm^2^) and JAK2 alone (151.500 nm^2^) were quite similar. This close resemblance suggests that ligand binding does not significantly affect the surface area accessible to the solvent, indicating that the hydrophilic and hydrophobic characteristics of the JAK2 protein are preserved during the interaction with MOL004112. Consequently, these results imply that the ligand binding does not induce major conformational changes that would alter residue exposure, thereby contributing to the stability of the protein–ligand complex.

**Figure 9:**
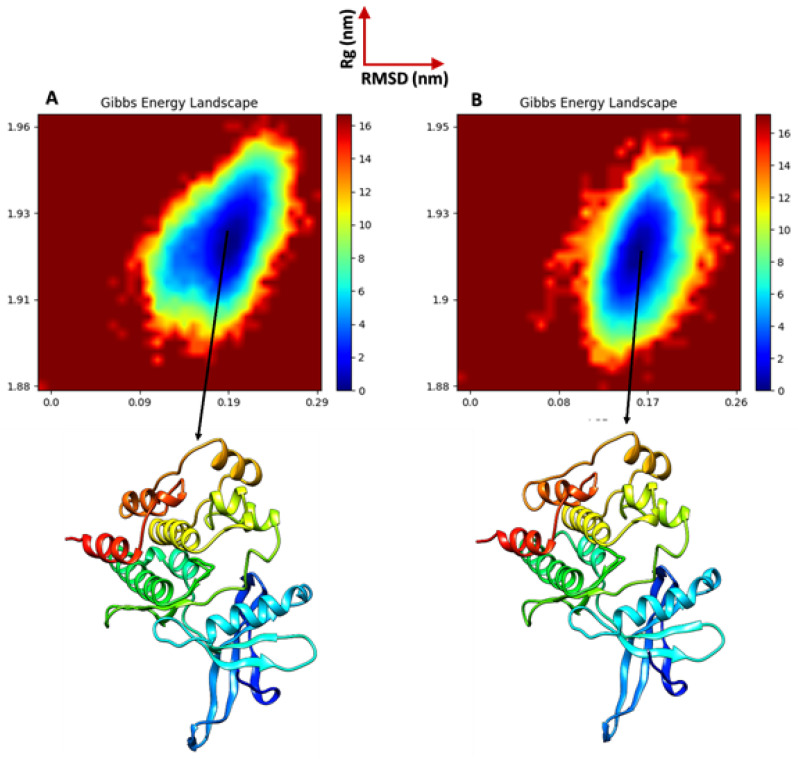
Free energy landscapes: (**A**) Free energy landscape of the JAK2-MOL004112 complex, illustrating the energy profiles associated with its conformational states and indicating the stability and potential folding pathways of the complex. (**B**) Free energy landscape of JAK2 alone, showcasing the energy profiles of its conformational states, providing insights into the stability and folding mechanisms of the protein without ligand interaction.

Molecular dynamics simulations are essential for modeling the free energy landscape (FEL) and analyzing the molecular folding behavior of proteins at an atomic level. In this study, the FEL of the JAK2-MOL004112 complex and JAK2 alone was constructed using the backbone atoms of JAK2 by integrating the root mean square deviation (RMSD) and the radius of gyration (Rg), both measured in nanometers. This approach facilitates a comprehensive understanding of the energy states associated with the conformational dynamics of the protein, providing insights into the stability and folding mechanisms of JAK2 in both its free and ligand–bound forms. The results of the free energy landscape (FEL) analysis are illustrated in Figure 9, which depicts the energy profiles linked to the conformational states of both the JAK2-MOL004112 complex and JAK2 alone, shedding light on the stability and potential folding pathways of the protein.

The visualization of the FEL enables a deeper understanding of the energetic barriers and favorable states that influence the molecular dynamics of the JAK2 protein in different conformational contexts. In the JAK2-MOL004112 configuration, the complex demonstrated conformational stability with a free energy value of ΔG = 0, corresponding to an RMSD of 0.19 nm and an Rg of 1.93 nm. In comparison, the JAK2-alone configuration exhibited the lowest free energy conformations, centered around an RMSD of 0.17 nm and an Rg of 1.92 nm. These results suggest that both the JAK2 protein and the JAK2-MOL004112 complex adopt stable conformations, with only minor differences in structural deviations and compactness, further supporting the stable interaction between JAK2 and the ligand.

## 3. Discussion

The antiviral properties of *Artemisia annua* L. have garnered significant interest due to its rich phytochemical profile and historical use in traditional medicine. This study revealed several potential antiviral targets by analyzing active compounds derived from *Artemisia annua*. Gene ontology (GO) term-enrichment analysis for the 19 common gene targets highlighted their involvement in critical immune processes, such as macrophage and NK cell proliferation, which are essential components of the antiviral immune response. These biological processes also include cardiac neural crest cell development, while cellular components, such as the IL-12/23 receptor complex and plasma membranes, play a central role in cell signaling. The involvement of these targets in innate and adaptive immune responses underlines their relevance in controlling viral infections, particularly through the modulation of immune signaling pathways. In terms of molecular functions, kinase activity, and more particularly, the activity of tyrosine kinases, was significantly enriched, highlighting their crucial role in regulating antiviral pathways. JAK/STAT signaling, activated by cytokines such as interferon, is a relevant example where JAK2 involvement is prominent in regulating antiviral immune responses. This has important implications in viral infections such as hepatitis B, influenza A, and potentially MERS-CoV, where the controlled activation of these pathways may help inhibit viral replication and modulate inflammation [30]. By focusing on key genes such as JAK2, our study highlights their central role in regulating immune responses, reinforcing the idea that these proteins represent promising therapeutic targets for future antiviral therapies [31,32]. Our docking simulations showed that the compound MOL001412 (Patuletin) binds more efficiently to JAK2 as compared to the reference ligand H36 and the approved drug Ruxolitinib. This compound demonstrated significantly stronger binding energies, with stable hydrophobic interactions and an increased number of hydrogen bonds formed with critical JAK2 residues. These results suggest a greater stability of ligand–protein complexes, which could contribute to a better inhibition of JAK2, a key regulator of antiviral responses via the JAK/STAT cascade. Furthermore, molecular dynamics simulations confirmed that these interactions are stable over a 200 ns time period, The minimal fluctuations in RMSD and RMSF indicate that MOL004112 binds to JAK2 without causing significant conformational changes. This stability is advantageous, as it may reduce the likelihood of adverse effects and improve the therapeutic efficacy of the compound. The slight increase in the radius of gyration (Rg) and solvent-accessible surface area (SASA) indicates that while ligand binding introduces some flexibility, it does not destabilize the protein’s structure, supporting the idea that the binding is both effective and stabilizing [33] and demonstrating that the JAK2 inhibition by Patuletin does not alter the overall protein structure. This stability is particularly important because it indicates that MOL001412 could maintain a long-lasting inhibition of JAK2, promoting a prolonged immune response against viruses. Therapeutically, JAK inhibitors (JAKi), such as Ruxolitinib, have demonstrated efficacy against several viral infections, including reducing excessive inflammation induced by dysfunctional immune responses [34]. Interestingly, the fact that MOL004112 does not cross the blood–brain barrier (BBB) may be a beneficial characteristic, particularly for treating peripheral viral infections without affecting the central nervous system’s functions. In contrast, Ruxolitinib’s ability to cross the BBB, along with its associated neurotoxicity risk, highlights the potential advantages of utilizing natural compounds like those from *A. annua* [7]. The development of antiviral therapies that selectively target peripheral sites of infection while minimizing central effects is a critical area of ongoing research [35].

Developing combination therapies that target both JAK2 and viral replication could offer a synergistic approach to enhance antiviral effects [36]. However, it is important to note that JAK/STAT signaling can have paradoxical effects in viral infections. While this pathway is crucial for antiviral defense, it can also facilitate viral replication in some cases, highlighting the need for fine regulation of this cascade. In conclusion, the results of this study show that MOL001412 is a promising candidate for the development of JAK2-targeted antivirals. Its potential efficacy in regulating immune responses, combined with its structural stability when bound to JAK2, makes it an attractive therapeutic option for viral infections such as MERS-CoV, hepatitis B, and influenza A. Future studies should focus on the in vivo evaluation of the antiviral activity of this compound to validate its clinical potential.

## 4. Materials and Methods

### 4.1. Bioactive Compounds and Potential Molecular Targets of A. annua

The active components of *A. annua* were selected from the Traditional Chinese Medicine Systems Pharmacological Analysis Database (TCMSP) (https://old.tcmsp-e.com/tcmsp.php, accessed on 4 July 2024). According to TCMSP, compounds with oral bioavailability (OB) greater than 30% and a drug-likeness (DL) greater than 0.18 are considered suitable for drug development. Based on these criteria, we filtered compounds using OB ≥ 30%, DL ≥ 0.18, and molecular mass between 250 and 500 Da. This resulted in eight identified active components of *A. annua*. The corresponding molecular targets of these compounds were identified using the SwissTarget Prediction database (https://swisstargetprediction.ch/, accessed on 6 July 2024). Duplicates were removed to yield potential targets for further investigation.

### 4.2. Potential Antiviral Targets

Potential antiviral therapeutic targets were gathered from the GeneCards database (https://www.genecards.org/, accessed on 6 July 2024) by using the keyword “antiviral”. This provided a list of candidate targets for studying the antiviral activity.

### 4.3. Design and Analysis of Protein Interaction Networks

We identified 19 genes of interest from the intersection of the *A. annua* targets and antiviral targets. Protein–protein interactions (PPI) were analyzed using the STRING 12.0 database (https://string-db.org/, accessed on 15 July 2024). The species selected was *Homo sapiens*, with a minimum interaction score of 0.4. The interaction network was exported as SIF files and visualized using Cytoscape 3.10.2 (https://cytoscape.org/, accessed on 3 October 2024), identifying hub genes based on their degree of connectivity.

### 4.4. Functional Analysis and Pathway Enrichment

Gene Ontology (GO) annotations and KEGG pathway enrichment were performed using DAVID (https://david.ncifcrf.gov/tools.jsp, accessed on 15 September 2024) and SHINY GO 0.80 (http://bioinformatics.sdstate.edu/go/, accessed on 20 September 2024). These tools helped identify biological processes and pathways relevant to the antiviral action of *A. annua* compounds.

### 4.5. Optimization of Protein Structures and Ligands for Docking Analysis

Protein–ligand docking was performed using the Schrödinger suite2018. The structure of JAK2 (PDB ID: 7F7W) was obtained from the Protein Data Bank. Protein preparation was performed using the Protein Preparation Wizard, including hydrogen atom addition, protonation state adjustments, and structural corrections. Ligands from *A*. *annua* were prepared with LigPrep2018, generating various conformations. Preliminary docking was conducted using Glide in Standard Precision (SP) mode, followed by Extra Precision (XP) docking for the top candidates. More details on the preparation methods for the protein and ligands can be found in our previous works [12,37,38]. The docking results were analyzed based on binding energy and interactions, such as hydrogen bonding and hydrophobic interactions. Discovery Studio software2021 (https://discover.3ds.com/discovery-studio-visualizer-download, accessed on 25 September 2024) was used for further interaction analysis.

### 4.6. Software and Tools for ADMET Analysis

Pharmacokinetic and toxicity predictions for the selected compounds were performed using ADMETlab 3 (https://admetlab3.scbdd.com/, accessed on 23 September 2024) and ProTox3 (https://tox.charite.de/protox3/, accessed on 23 September 2024). These tools provided insights into the absorption, distribution, metabolism, excretion, and toxicity properties of the compounds.

### 4.7. Molecular Dynamics Simulations (MDS)

Molecular dynamics simulations were performed on the JAK2-ligand complex (MOL004112_7F7W) and the reference protein (7F7W) using the Gromacs-2023 package for 200 ns. The SwissParam webserver [39] was used to generate topology parameters for the ligands, while the CHARMM27 all-atom force field was employed for the protein. The simulation system was solvated using the TIP3P water model, and Na^+^ and Cl^−^ ions were added to neutralize the system. Energy minimization was conducted using the steepest descent algorithm [40,41].

Following energy minimization, equilibration was performed in two phases: NVT (constant number of particles, volume, and temperature) for temperature stabilization and NPT (constant number of particles, pressure, and temperature) for pressure stabilization, both at 300 K. The root mean square deviation (RMSD), root mean square fluctuation (RMSF), the radius of gyration (Rg), and the solvent accessible surface area (SASA) were calculated to evaluate the system’s stability and dynamics. The free energy landscape (FEL) analysis was performed using RMSD and Rg as reaction coordinates [42].

## 5. Conclusions

This study highlights the antiviral potential of *Artemisia annua* L. through an integrated network pharmacology and molecular docking approach. We identified several active compounds, including MOL004112 (Eupatin), and key therapeutic targets, such as JAK2, which play crucial roles in modulating immune and antiviral responses. Protein–protein interaction (PPI) networks and enrichment analyses revealed important pathways and mechanisms that could be exploited in the development of novel antiviral treatments. Additionally, ADME profiling and molecular dynamics simulations confirmed the stability of MOL004112, indicating its potential as a candidate for future research.

These findings open new perspectives for utilizing *Artemisia annua* in combating viral infections and underscore the importance of further studies into its mechanisms of action. The complex formed between the identified molecule and JAK2 maintained structural stability throughout an extensive 200 ns molecular dynamics simulation, indicating that while MOL004112 effectively inhibits the biological activity of JAK2, it does so without inducing significant conformational changes or compromising the protein’s structural integrity. Such results suggest that the inhibitory effect is achieved through a mechanism that does not disrupt the overall architecture of JAK2, reinforcing the idea of a stable and non-disruptive interaction between the molecule and the protein.

In summary, this research contributes to our understanding of the antiviral properties of *Artemisia annua* and emphasizes the importance of investigating drugs derived from natural compounds for the treatment of viral infections.

## Figures and Tables

**Table 1:** Summary of *Artemisia* compounds selected from TCMSP.

Mol. ID	Molecule Name	MW	(%) OB	DL (%)
MOL002235	EUPATIN	360.34	50.8	0.41
MOL004112	Patuletin	332.28	53.11	0.34
MOL007404	vitexin_qt	270.25	52.18	0.21
MOL007389	artemisitene	280.35	54.36	0.31
MOL007415	[(2S)-2-[[(2S)-2-(benzoylamino)-3-phenylpropanoyl]amino]-3-phenylpropyl] acetate	444.57	58.02	0.52
MOL007423	6,8-di-c-glucosylapigenin_qt	270.25	59.85	0.21
MOL007425	dihydroartemisinin	284.39	50.75	0.3
MOL007426	deoxyartemisinin	266.37	54.47	0.26

**Table 2:** Enrichment results: number of genes, pathway genes, enrichment scores, and associated pathways.

Enrichment FDR	nGenes	Pathway Genes	Fold Enrichment	Pathway	Genes
1.45 × 10^−9^	5	79	160.90	Path:hsa01521 EGFR tyrosine kinase inhibitor resistance	JAK1 JAK2 MAPK1 MAPK3 RAF1
1.06 × 10^−7^	4	70	145.27	Path:hsa04917 Prolactin signaling pathway	JAK2 MAPK1 MAPK3 RAF1
2.22 × 10^−9^	5	89	142.82	Path:hsa05235 PD-L1 expression and PD-1 checkpoint pathway in cancer	JAK1 JAK2 MAPK1 MAPK3 RAF1
2.25 × 10^−9^	5	92	138.17	Path:hsa04658 Th1 and Th2 cell differentiation	JAK1 JAK2 MAPK1 MAPK3 TYK2
1.34 × 10^−10^	6	112	136.19	Path:hsa05145 Toxoplasmosis	HSPA8 JAK1 JAK2 MAPK1 MAPK3 TYK2
1.17 × 10^−7^	4	76	133.80	Path:hsa05140 Leishmaniasis	JAK1 JAK2 MAPK1 MAPK3
1.17 × 10^−7^	4	76	133.80	Path:hsa05212 Pancreatic cancer	JAK1 MAPK1 MAPK3 RAF1
4.46 × 10^−9^	5	108	117.70	Path:hsa04659 Th17 cell differentiation	JAK1 JAK2 MAPK1 MAPK3 TYK2
5.91 × 10^−10^	6	162	94.16	Path:hsa05161 Hepatitis B	JAK1 JAK2 MAPK1 MAPK3 RAF1 TYK2
5.91 × 10^−10^	6	171	89.20	Path:hsa05164 Influenza A	JAK1 JAK2 MAPK1 MAPK3 RAF1 TYK2
1.64 × 10^−8^	5	143	88.89	Path:hsa04550 Signaling pathways regulating pluripotency of stem cells	JAK1 JAK2 MAPK1 MAPK3 RAF1
6.66 × 10^−7^	4	120	84.74	Path:hsa04935 Growth hormone synthesis secretion and action	JAK2 MAPK1 MAPK3 RAF1
2.37 × 10^−8^	5	157	80.96	Path:hsa05160 Hepatitis C	JAK1 MAPK1 MAPK3 RAF1 TYK2
7.29 × 10^−7^	4	126	80.70	Path:hsa04380 Osteoclast differentiation	JAK1 MAPK1 MAPK3 TYK2
9.54 × 10^−10^	6	194	78.62	Path:hsa05167 Kaposi sarcoma-associated herpesvirus infection	JAK1 JAK2 MAPK1 MAPK3 RAF1 TYK2
4.17 × 10^−8^	5	179	71.01	Path:hsa05152 Tuberculosis	JAK1 JAK2 MAPK1 MAPK3 RAF1
1.06 × 10^−7^	5	223	57.00	Path:hsa05208 Chemical carcinogenesis-reactive oxygen species	HMOX1 MAPK1 MAPK3 PTPN1 RAF1
5.86 × 10^−7^	5	331	38.40	Path:hsa05165 Human papillomavirus infection	JAK1 MAPK1 MAPK3 RAF1 TYK2
7.29 × 10^−7^	5	354	35.90866290	Path:hsa04151 PI3K-Akt signaling pathway	JAK1 JAK2 MAPK1 MAPK3 RAF1
1.14 × 10^−7^	6	530	28.781132075	Path:hsa05200 Pathways in cancer	HMOX1 JAK1 JAK2 MAPK1 MAPK3 RAF1

**Table 3:** XP Docking scores of *A. annua* compounds with the JAK2 protein.

Compounds	XP Score Kcal/Mol
Mol007426	−4.651
Mol004112	−10.50
Mol007389	−4.814
Mol007404	−8.732
Mol007415	−7.552
Mol007423	−7.494
Mol007425	−4.580
Mol002235	−9.570
36H	−9.432
(drug) Ruxolitinib	−6.829

**Table 4:** Detailed analysis of the interactions and bindings of the two best compounds with the JAK2 protein (7F7W).

Compounds	H-Bond	Number	Hydrophobic	Number	Other
Mol002235	LYS581 Gln626 Val629 Lys630 Asn678	7	Ile559	10	/
			Leu572		
			Leu551		
			Leu680		
Mol004112	Leu551 Val629	8	Ile559	8	/
	Lys630 Gln626 Asn678 Lys581		Leu572		
			Leu551		
			Leu680		
H36	Val629 Lys581 Ser550	5	Ile559 Lys677 Leu579 Val610 Leu680	8	Leu551
*Ruxolitinib* (drug)	Thr636 Gln626 Glu627	3	Leu549 Val629 Leu680 Leu551	8	/

**Table 5:** Molecular property prediction using ADMELAB.2.

Compounds	MW	nRot	nHet	Flexibility	TPSA	Nring	logS	logP
MOL004112	332.05	2	8	0.111	140.59	3	−3.616	1.428
MOL002235	360.08	4	8	0.222	115.59	3	−3.79	1.777
Ruxolitinib	306.16	4	6	0.19	83.18	5	−3.246	2.392

**Table 6:** Medicinal chemistry properties prediction using ADMELAB.2.

Compounds	QED	SAscore	Pfizer Rule	Lipinski Rule	PAINS	Golden Triangle
MOL004112	0.449	2	0	0	0	0
MOL002235	0.65	2	0	0	0	0
Ruxolitinib	0.8	3	0	0	0	0

**Table 7:** Absorption and distribution predictions using ADMELAB.2.

Compounds	Caco-2 Permeability	Pgp-Inhibitor	HIA	PPB	Vd
MOL004112	−5.536	no	excellent	97.665	−0.74
MOL002235	−5.306	yes	medium	97.801	−0.69
Ruxolitinib	−5.158	yes	excellent	51	0.04

**Table 8:** Description of compound MOL004112.

**MOL004112**	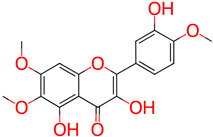
IUPAC	2-(3,4-Dihydroxyphenyl)-3,5,7-trihydroxy-6-methoxy-4-benzopyrone
Other Identifiers	PubChem CID5281678 CHEMBL77588
FORMUL	C_16_H_12_O_8_
SYNONYM	Patuletin
SMILE	COC1=C(C2=C(C=C1O)OC(=C(C2=O)O)C3=CC(=C(C=C3)O)O)O
Chemical Classes	Flavonoids
Activity biologic	anti-inflammatory [23], antiviral [24,25,26], anticancer [27,28]

**Table 9:** Average values of RMSD, RMSF, Rg, and SASA.

Metric	JAK2_mol004112	JAK2 Alone
RMSD (nm)	0.300	0.199
RMSF (nm)	0.150	0.101
Rg (nm)	1.972	1.919
SASA (nm^2^)	148.313	151.500

## Data Availability

Data are contained within the article.

## Supplementary Materials

The following supporting information can be downloaded at: https://www.mdpi.com/article/10.3390/ph17111539/s1, Figure S1: Chemical Structures of Selected Compounds (Artemisia); Figure S2:Correlation Analysis Between Ki and XP Score: Insights into Binding Affinity and Inhibitory Potency; Figure S3: Validation of Docking Results Using RMSD Analysis from DockRMSD; Figure S4: 2D Interaction of Compound 36H with JAK2 Protein (PDB ID: 7F7W); Figure S5: 2D Interaction of Ruxolitinib with JAK2 Protein (PDB ID: 7F7W); Table S1: List of Genes Associated with Artemisia and Antiviral Activity, Including Common Genes; Table S2: Binding Affinity and Inhibitory Potency of Compounds; Table S3: In silico prediction of metabolism and excretion; Table S4: In silico prediction of Toxcity.
