# Supplementary material for: Exploring the Antiviral Potential of Artemisia annua Through JAK-STAT Pathway Targeting: A Network Pharmacology Approach
Source: Pharmaceuticals (Basel). 2024 Nov 16;17(11):1539. doi: 10.3390/ph17111539 (PMC11597232; doi:10.3390/ph17111539)
Supplement: Supplementary file 1 [file pharmaceuticals-17-01539-s001.zip › pharmaceuticals-3292320-supplementary.pdf]

Article

Exploring the Antiviral Potential of *Artemisia annua* Through JAK-STAT Pathway Targeting: A Network Pharmacology Approach

Mebarka Ouassaf [1,*], Lotfi Bourougaa[1], Farial Bahaz [2] , and Bader Y. Alhatlani [3,**]

[1] Group of Computational and Medicinal Chemistry, LMCE Laboratory, University of Biskra, BP 145 Biskra, 07000, Algeria. M.O: nouassaf@univ-biskra.dz. L.B: lotfi.bourougaa@univ-biskra.dz

[2] Laboratory of Organic Materials and Heterochemistry, Echahid Cheikh Larbi Tebessi University, Tebessa, Algeria. F.B: farial.bahaz@univ-tebessa.dz,

[3] Unit of Scientific Research, Applied College, Qassim University, Buraydah 52571, Saudi Arabia. B.A: balhatlani@qu.edu.sa

* Correspondence: balhatlani@qu.edu.sa; nouassaf@univ-biskra.dz.

Figure S1: Chemical Structures of Selected Compounds (Artemisia)

COMPOUNDS
**MOL004112** 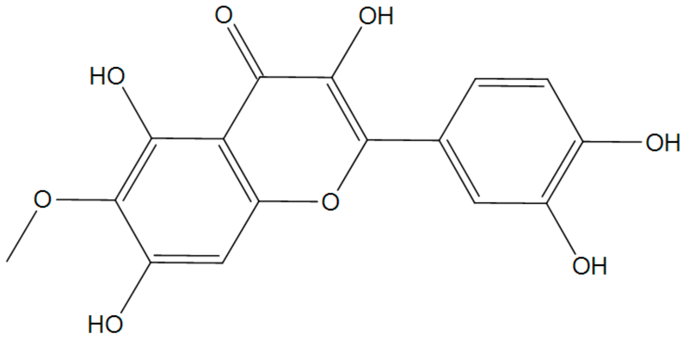

MOL002235

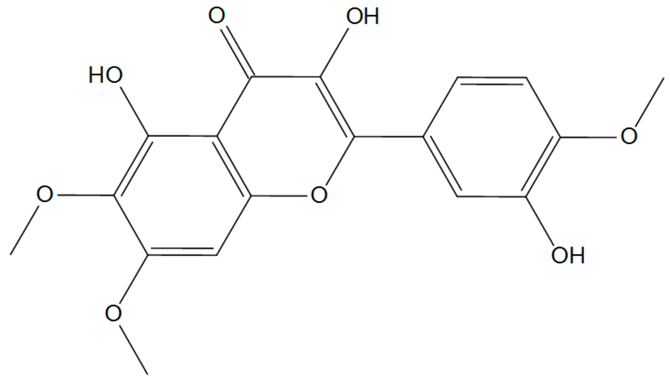

MOL007425

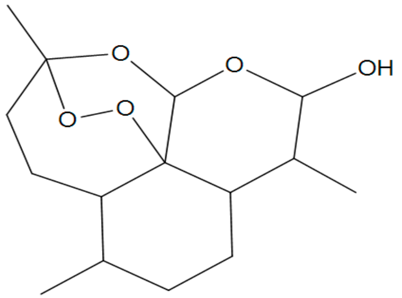

MOL007404

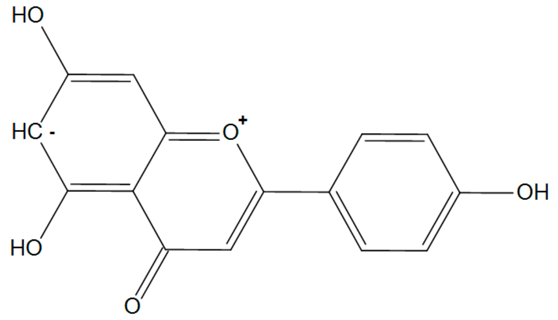

MOL007389

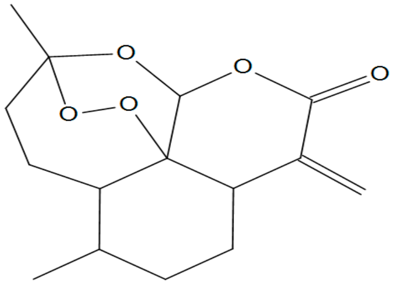

MOL007423

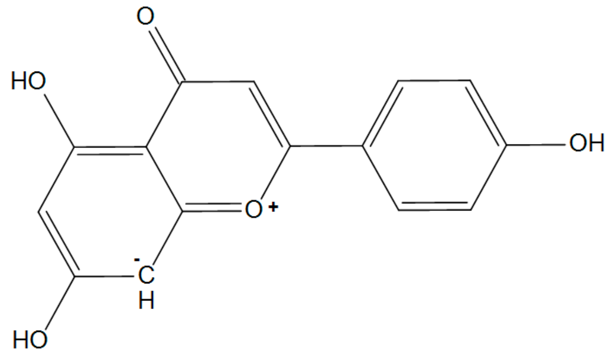

MOL007426

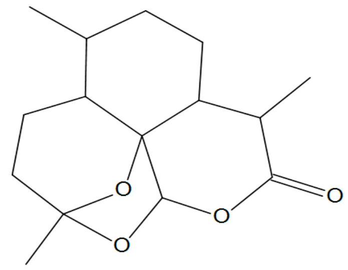

MOL007415

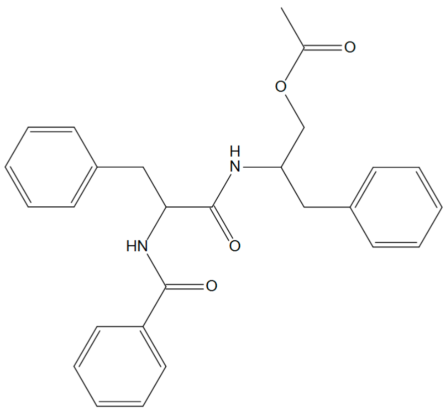

Figure S2: Correlation Analysis Between Ki and XP Score: Insights into Binding Affinity and Inhibitory Potency

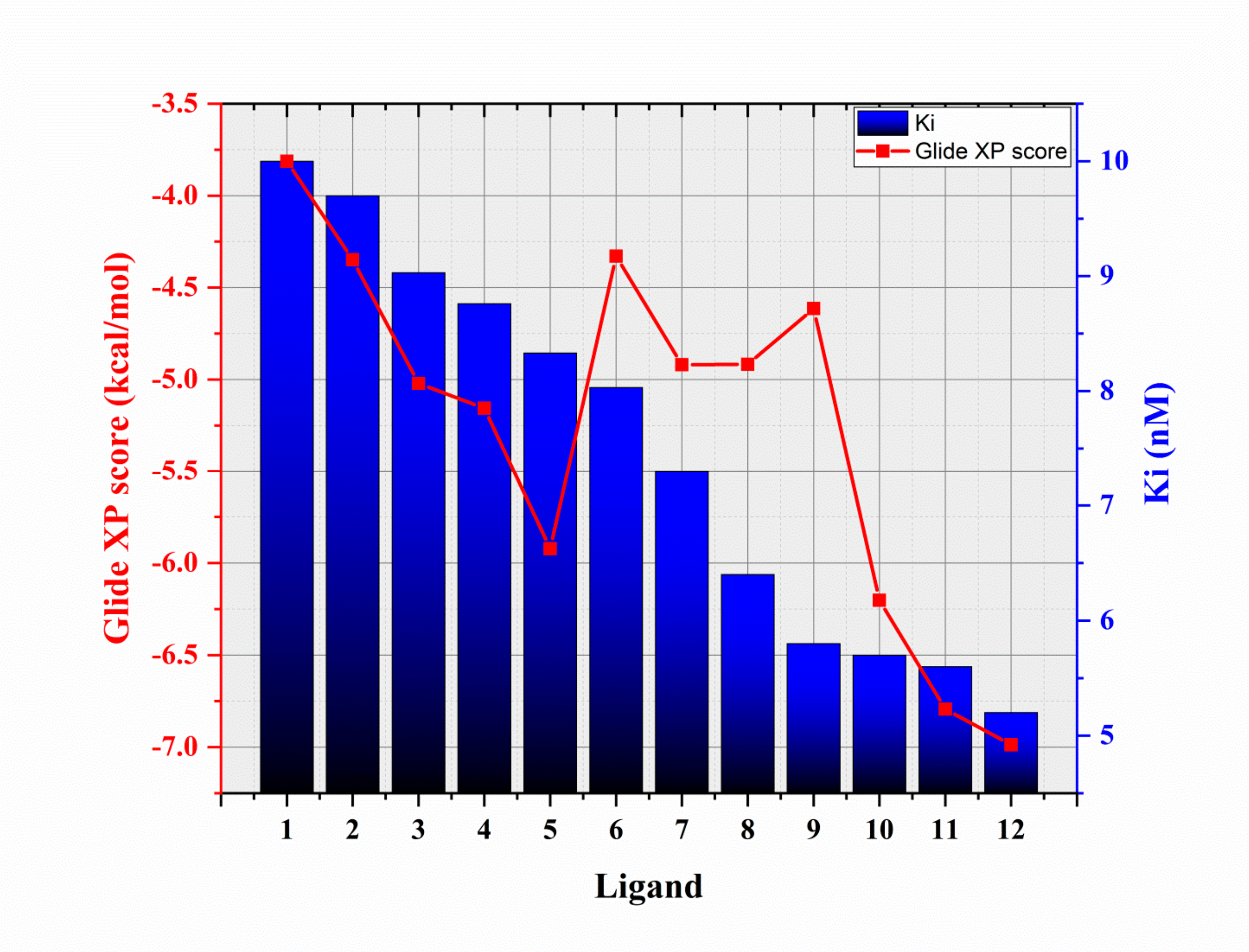

```
calculated Docking RMSD: 2.073

Total # of Possible Mappings: 256
Optimal mapping (First file -> Second file, * indicates correspondence is not one-to-one):
N  1 -> N  1
C  2 -> C  2
C  3 -> C  3
C  4 -> C  4
C  5 -> C 25 *
C  6 -> C 24 *
C  7 -> C  7
C  8 -> C  8
C  9 -> C  9
C 10 -> C 12 *
C 11 -> C 11
C 12 -> C 10 *
N 13 -> N 13
C 14 -> C 14
C 15 -> C 15
C 16 -> C 16
N 17 -> N 17
C 18 -> C 18
N 19 -> N 19
C 20 -> C 20
C 21 -> C 21
C 22 -> C 22
N 23 -> N 23
C 24 -> C  6 *
C 25 -> C  5 *
N 26 -> N 26
C 27 -> C 27
O 28 -> O 28
F 29 -> F 29
C 30 -> C 30
N 31 -> N 31
C 32 -> C 32
```

```
C 33 -> C 33
C 34 -> C 34
```

Figure S3: Validation of Docking Results Using RMSD Analysis from DockRMSD

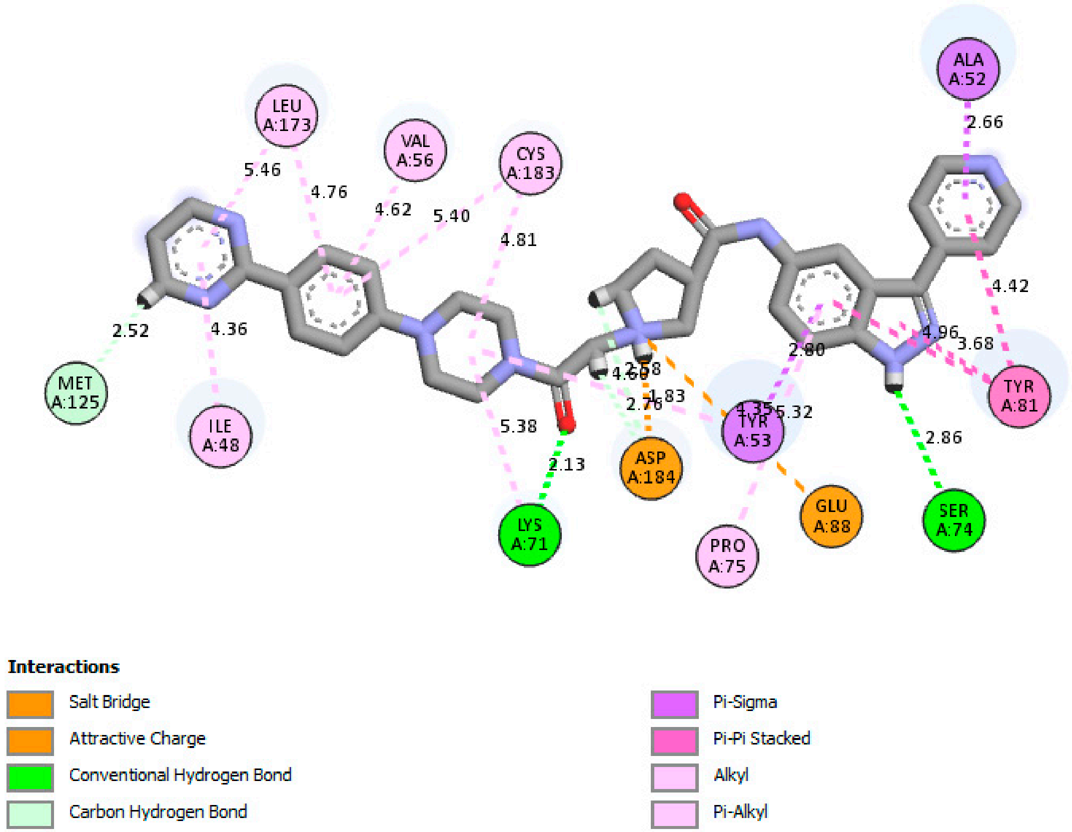

FigureS4: 2D Interaction of Compound 36H with JAK2 Protein (PDB ID: 7F7W)

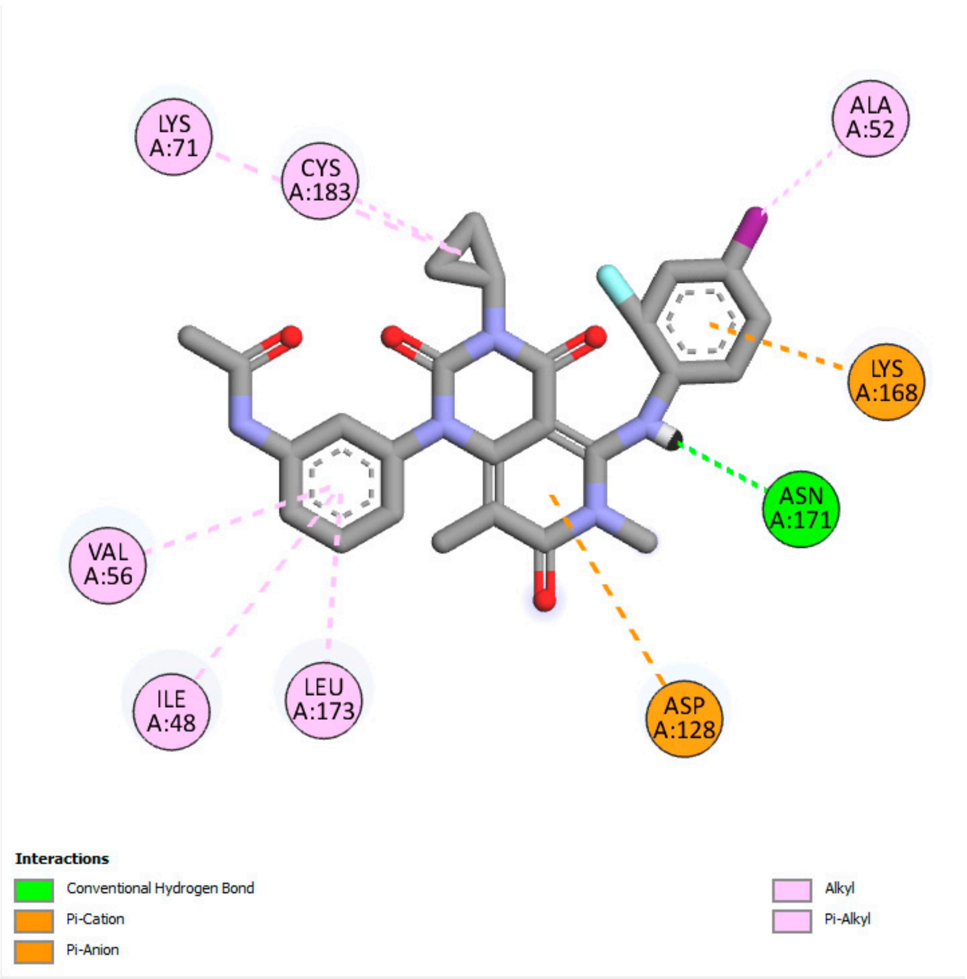

Figure S5: 2D Interaction of Ruxolitinib with JAK2 Protein (PDB ID: 7F7W)

Table S1: List of Genes Associated with Artemisia and Antiviral Activity, Including Common Genes.

Names	total	elements
antiviral Artemisia	19	P03956 O60674 P11142 P28482 P25105 P18031 P06493 Q9NYA1 Q9UHD2 P04049 P08254 Q13554 P27361 P10636 P23458 Q9UPY5 Q93009 P09601 P29597
Artemisia	362	Q92847 O00408 Q9HBH9 O95931 P37268 P55085 P23975 P27986 P11926 P18054 P21980 O60218 O60285 P08473 P17948 Q15759 P43088 Q9UBN7 P53779 O14746 P30989 P05093 P08173 P35354 P12821 O43570 O14649 P07451 P18825 Q99500 P11362 P36544 Q15056 P16581 P53582 Q9UNA0 P34995 P11309 P04629 P14679 P09917 P17936 P09874 P49286 P35869 Q92769 P02766 P05089 P14780 Q12884 P31645 P21397 O00329 P22748 P19021 P48147 P32239 P15907 P41145 P09237 P09467 P16050 P19099 O60706 P10275 Q9NZ42 Q53GL7 P20309 Q08493 Q07820 P48039 P49354 P49356 Q13093 P03372 P53355 P14867 P37059 P28161 P28472 P18507 P31644 Q9H3R0 P43235 P50416 P42336 P27986 P17516 P23280 Q16539 P35968 Q02750 P08514 P05106 Q15746 P52333 P17252 P31749 P11474 Q01959 P00533 P30530 Q16850 P30305 P26358 P21731 P28074 P11217 P35372 P16109 P49840 Q14432 Q02127 O75874 P25025 Q9NPH5 P06737 P29466 Q01432 P20701 P05362 P05107 Q8N1Q1 P53350 P11021 P36888 P04626 P05186 P11712 P16473

		Q9NPC2 Q13936 P10721 P22894 P07858 P51449 P25103 P11511 O00519 Q8TDS4 P28472 P34903 P18507 Q15858 Q13510 Q05397 P23141 O76074 P47989 Q08499 P41594 P51812 P55072 P12931 P29372 Q9UNQ0 P20292 B2RXH2 P08253 O15151 P11473 P33527 P42330 Q99808 P24593 P23526 P47869 P28472 P18507 P62942 Q99538 P08069 O60930 P56192 O14684 P09619 P43490 Q9H2K2 P25774 P10635 Q13332 P25100 O60911 P30518 O00257 Q9ULX7 Q16853 Q16665 P00734 P22001 P08684 Q06418 P61586 P49841 P09211 P25024 P00918 P05129 P78536 P13866 Q9HC97 P45983 Q92731 Q16790 P42338 P14061 P43405 Q16198 P52333 P23458 O95819 Q14790 P04054 Q16875 Q9NWZ3 P78527 P30556 O60341 P08238 Q92793 Q6PCB7 Q16678 Q16512 P35610 P34998 P45880 P23946 O75173 P20618 P14550 P51677 P55210 Q9UM73 O75164 Q96GD4 Q96RR4 P56817 P28845 P08912 Q9NY91 P22303 P11229 P23219 P05177 P15121 Q00534 Q14654 O60706 P15538 P49137 P30559 P25101 P35218 P52895 P01112 P68400 P27338 P48544 P48549 P21964 Q96S37 P0DMS8 P0DMS9 P49810 Q9NZ42 Q92542 Q96BI3 P49768 Q8WW43 P45984 P07099 P29474 P11940 P29274 Q96RI1 P05164 Q9HC52 P15309 P32249 P31639 P37231 P23416 P00491 P04818 P30838 Q9HC98 P43166 P24941 P78396 P20248 P15382 P51787 Q99685 P27695 P42892 P15090 P14174 P36897 P54577 P43220 Q9NRA0 Q07343 P08172 P11597 P00488 P14151 P30542 P48736 Q13547 P05067 Q04828 Q15149 P04229 P11388 P49336 P07711 Q8WWL7 P06493 P14635 O95067 P42336 P45452 O15530 P39900 Q13627 P04062 P28472 P18507 P14867 P22735 Q13639 P00742 P21452 Q14289 P16083 P08236 P07384 P24941 P09960 P51955 P08183 P33261 P49682 P06213 P22460 P39086 P23415 P00747 Q9Y5Y4 O14965 P24530 P15056 P35228 Q04760 Q495T6 P04035 P29275 P14867 P47870 P18507 P28907 O00748 P55263 Q9Y233 Q15078 Q00535 P49721 O00311 P16234 Q9UDY8 P50579 O95271 Q9HAS3 P35813 P11387 O60725 P00915 P14555 Q5S007 P31644 P41143 P00918 Q96RJ0 P07900 Q99720 P21917 Q9H3N8 P08311

		P34972 P07333 P41143 P08581 P32247
antiviral	335	Q9NVI7 Q9H0E7 P05161 P02778 P19793 P01574 O00337 Q9H8W5 Q9BYF1 Q13325 Q9Y2K6 O43432 Q8WXG1 Q8NFH4 O14920 Q14134 Q9BVG3 Q8NFH5 Q3ZCM7 P01570 A8MT69 Q14457 Q6L8Q7 A9QM74 P32881 P48506 P17181 Q8N2Z9 P48507 Q96PP8 Q9H8X9 Q12905 P04637 P00973 P49790 Q6PD62 Q9BYJ4 Q9HC16 P30153 P17693 P32455 P33402 O60573 Q6PJ69 Q71U36 Q86WT6 P29459 Q16236 P62979 P12268 P55735 P14316 O15287 P01911 Q9UPQ4 P05198 Q9BUF5 Q99567 P41226 Q9P243 Q9BQF6 P01375 Q14142 P61769 Q14145 P23497 P01563 P60842 P19474 P35232 Q9Y239 Q9Y4X5 P52948 Q8WTX9 Q8IUC6 P25963 Q9BZR9 P01889 P01566 O15344 Q96HA1 Q9Y4K3 Q7Z434 Q06124 P52564 Q9NY65 Q9UII4 O75569 P67809 P12314 Q15172 Q92667 P38484 P55265 O75369 Q9C029 P33076 P32456 P10914 P62987 O00635 O75525 P46934 P05362 P09912 P19525 P01909 P13500 O00505 P80217 P0CG48 Q15646 P12270 P61221 O15131 Q9Y2G2 P01567 P19174 P37198 P15260 P30511 Q9Y3Z3 O15105 Q9Y577 P16333 Q13237 P01579 Q13526 Q96C10 Q04637 Q86WV6 P04439 P15559 Q9UQM7 P01571 Q05655 P29350 Q8WVC0 Q14974 Q6NTF7 P13762 Q9UMW8 P34931 P08949 P68366 Q14149 P13164 Q5SRE5 Q14653 P01562 Q01628 P10145 P05015 Q15633 P29728 Q9C030 Q9Y5Z7 Q14164 Q6NZ36 Q9NX74 P30154 Q00978 Q8N1F7 P05538 P0CG47 P09913 P05014 Q9BQE3 Q9BW27 P33908 Q96B02 O15360 P40305 O14879 Q02556 P52630 Q0VG06 Q9UHH9 O15111 O43143 P78406 P52294 P0DMV8 Q6P1J9 Q13885 P28336 P05231 P0DPH8 Q9BVA1 Q96AZ6 Q9BVL2 P63279 Q7Z4K8 Q6PEY2 Q04206 Q9NPI8 Q30154 Q2M2I8 Q5D1E8 Q13568 Q9BTP7 Q12906 P13284 O75925 P35658 Q9NUL5 P48551 Q01629 Q14240 Q9H4B7 P20592 Q9H078 P63165 P06730 Q9UDY6 Q9H171 P22681 Q10589 Q9C040 Q96H79 Q13217 P67775 P40855 A6NHL2 P01569 P13747 P28062 Q92985 A0PJW8 Q13555 P19838 P40763 Q9NW38 Q9Y6K5 Q05823 Q9BYX4 P57740 Q00597 Q14258 Q6GPH4 P20042 P01906 P78344 P19320 O15504 Q96PP9 Q99623 Q9C035 P0DMV9 O95786 O00629 Q9HB96 P01568 P13591 Q9BTX1 Q15306 P20839 Q8IUD6 O15524 Q8NB91 Q8N7H5 P49792 P01903 O75382 Q6ZN66 P29590 P20591 Q9Y6K9 P09210 Q12769 Q9UKX7 P06748 P61088 Q8N5X7 Q8NFH3 Q8N8V2 P51965 P16070 Q7Z3B4 Q02153 P04350 Q9P0N8 Q13509 P68363 Q96EE3 P52292 Q8TEM1 P0DPH7 A4D0T7 P11226 P21333 P54652 P38919 P10321 O14543 Q8IYD8 P59044 Q8IWZ4 Q8WUM0 Q9NRG9 Q02108 Q9BZY9 P01920 O75343 Q12899 P79483 P09914 Q9C000 O75688 P41091 P17706 P02795 Q16222 P62714 P42224 O14933 Q9H0R5 Q8IYM9 P20036 P05013 O14896 Q9Y5S8 P37840 O75694 P18146 Q7Z2W4 P68371 P01584 O60231 Q92621 Q08211 Q13557 Q6AZZ1 P04440

Table S2: Binding Affinity and Inhibitory Potency of Compounds:

Compounds	Cid Pubchem	Ki (nM)	SCORE XP (Kcal/Mol)
**1**	53341248	10	3.813-
**2**	70497750	9.7	4.348-
**3**	118522328	9.03	5.021-
**4**	118522459	8.76	5.156-
**5**	118522732	8.33	5.923-
**6**	118522622	7.30	4.920-
**7**	60170769	6.4	4.917-
**8**	88710224	5.7	6.202-
**9**	70497097	5.6	6.793-
**10**	71450071	5.2	6.987-

Table S3 In silico prediction of metabolism and excretion

Metabolism	MOL004112	MOL002235	Ruxolitinib
**CYP1A2 inhibitor**	yes	yes	non
**CYP2C19 inhibitor**	non	yes	non
**CYP2C9 inhibitor**	non	yes	non
**CYP2D6 inhibitor**	non	non	non
**CYP3A4 inhibitor**	non	non	non
excretion			
**CL**	0,975	4.086	5,932
**T1/2**	1,557	1.734	1,339

Table S4 In silico prediction of TOXCITY

compounds	Hepatotoxicity	Neurotoxicity	Immunotoxicity	Mutagenicity	Cytotoxicity	BBB barier	Ecotoxicty
Mol002114	non	non	non	non	non	non	non
Mol002235	non	non	non	non	non	non	non
Ruxolitinib	non	yes	non	non	non	yes	yes
